# Does Embodiment of Verbs Influence Predicate Metaphor Processing in a Second Language? Evidence From Picture Priming

**DOI:** 10.3389/fpsyg.2021.759175

**Published:** 2021-11-03

**Authors:** Yin Feng, Rong Zhou

**Affiliations:** ^1^School of Foreign Studies, South China Normal University, Guangzhou, China; ^2^Center for Language Cognition and Assessment, South China Normal University, Guangzhou, China

**Keywords:** predicate metaphor, embodiment, sensorimotor, L2 proficiency, metaphor novelty, indirect categorization, intermediate entity

## Abstract

Distinct from nominal metaphors, predicate metaphors entail metaphorical abstraction from concrete verbs, which generally involve more action and stronger motor simulation than nouns. It remains unclear whether and how the concrete, embodied aspects of verbs are connected with abstract, disembodied thinking in the brains of L2 learners. Since English predicate metaphors are unfamiliar to Chinese L2 learners, the study of embodiment effect on English predicate metaphor processing may provide new evidence for embodied cognition and categorization models that remain controversial, and offer practical insights into L2 metaphor processing and pedagogy. Hence, we aim to investigate whether the embodiment of verbs, via the activation of sensorimotor information, influences two groups of L2 learners during their comprehension of conventional and novel predicate metaphors. The results show a significant effect of embodiment: a stronger facilitation for novel predicate metaphors in both higher-level and lower-level groups, and a weaker facilitation for conventional predicate metaphors in the lower-level group. The findings demonstrate preliminary evidence for a graded effect of embodiment on predicate metaphors processing, modulated by L2 proficiency and metaphor novelty. The study supports a hybrid view of embodied cognition and reveals that sensorimotor aspects of verbs may be the intermediate entity involved in the indirect categorization.

## Introduction

Metaphorical language is not just a poetic expression but also a conceptual device to communicate abstract ideas. The majority of studies in the past decades have focused on nominal metaphors in the typical “*A is B*” structure (e.g., *The rumor was a virus*), but few have been conducted on predicate metaphors (e.g., *The media bent the truth*), despite their frequent occurrence in daily discourses (Cameron, 2008; Steen et al., 2010; Goatly, 2011). Compared with literal sentences that convey physical senses (e.g., *The repairman bent the pipe*), predicate metaphors use verbs figuratively via abstraction from concrete action terms. Recently, neural studies found sensorimotor activations across brain regions when English natives read predicate metaphors (Chen et al., 2008; Desai et al., 2013; Obert et al., 2014; Lai et al., 2019); however, it has not been well-understood how action-based metaphors go beyond embodiment to create abstract thoughts in the brains of L2 learners whose language proficiency levels could affect difficulties of comprehension and embodiment. Furthermore, predicate metaphors comprehension may be a process of indirect categorization with an intermediate entity (Utsumi and Sakamoto, 2011; Obert et al., 2018), but its processing mechanisms in native language and second language were not fully studied. The aims of the present study are thus to investigate whether and how the embodiment of verbs, via the activation of sensorimotor properties, influences two groups of L2 learners during their comprehension of conventional and novel predicate metaphors and to explore the issues of intermediate entity in their processing of predicate metaphors.

A predicate metaphor is a linguistic construction that exemplifies the embodied nature of cognition since verbs generally entail more action content. Embodied cognition (Lakoff and Johnson, 1980; Glenberg and Kaschak, 2002; Gibbs, 2006a; Zwaan and Taylor, 2006; Estes et al., 2008; Glenberg, 2010) holds that cognition is grounded in the physical body and sensorimotor system. Abstract concepts can be understood via metaphors, which link abstract, less familiar knowledge with concrete and familiar experience. For example, reading tactile metaphors (e.g., *a rough day*) and taste metaphors (e.g., *a sweet compliment*) can activate sensory regions responsive to touch and taste (Lacey et al., 2012; Citron and Goldberg, 2014). Reading action metaphors related to motion content (e.g., *bent the truth*) can activate regions involved in motor perception (Desai et al., 2011, 2013; Lauro et al., 2013). The stronger view of embodied accounts (Gibbs et al., 2004; Gibbs, 2006b) believes that metaphors are comprehended *via* sensorimotor simulation, just as literal language is comprehended; while the weaker view (Boroditsky and Ramscar, 2002; Cardillo et al., 2012; Jamrozik et al., 2016) argues that only novel metaphors draw on sensorimotor information from the source domain, but the representation of source domain becomes abstracted as metaphoric use increases. A predicate metaphor is a typical vehicle to address these mixed views as it allows a verb to be used literally to describe physical action and metaphorically to convey abstract ideas.

Recent neural studies have supported the embodied views of predicate metaphor processing. For example, a few secondary motor regions were found to be involved when participants read familiar predicate metaphors, but the primary sensory and motor regions were more activated when they read unfamiliar novel metaphors (Desai et al., 2011, 2013; Cardillo et al., 2012), suggesting that novel predicate metaphors relied more on sensorimotor information corresponding to the verbs. However, other studies showed inconsistent motor activations in the brain (Watson et al., 2013). It seems that novelty or conventionalization of metaphors plays a role in the mixed results on embodiment. According to the career of metaphor theory (Bowdle and Gentner, 2005), all metaphors start as novel expressions, then evolve into conventional expressions, and eventually become literal expressions that lose the figurative meaning. For predicate metaphors, the figurative meaning centers on the concrete motion aspect of verbs. When predicate metaphors (e.g., *kick the habit*) are conventionalized over time, the salient meanings with deep relational alignment will remain in the brain (e.g., *give up something*), but the surface sensorimotor properties of the base meaning will be shed (e.g., *move the foot forward*). In this sense, there may be a graded embodiment for metaphor processing relative to metaphor novelty (Desai et al., 2013; Jamrozik et al., 2016).

In the past decade, many behavioral studies have addressed the embodied qualities of verbs (Richardson et al., 2001, 2003; Wilson and Gibbs, 2007), yet few studies have studied predicate metaphors in a sentence context or discourses, and it remains unclear what type of sensorimotor activation (e.g., visual or auditory) could influence predicate metaphor comprehension. For the structure of word meaning, a major division lies between the conceptual structure and spatial structure, the latter of which involves perception and action (Jackendoff, 2002); therefore, when a picture denoting an action is presented, the sensorimotor elements in the spatial structure are likely to be activated before a particular verb meaning is accessed in the conceptual structure. The imagery of such an action in the spatial structure can be readily available without retrieving a verb because the processing systems of verbal stimuli (text) and non-verbal stimuli (image) are functionally and structurally independent (dual coding theory, Paivio, 2014). In this sense, if one relies on the sensorimotor system when reading predicate metaphors, the imagery (i.e., embodiment) may facilitate abstraction from concrete meaning and result in faster comprehension.

Relatively few accounts have been proposed to explain how predicate metaphors are comprehended. Based on the studies on nominal metaphors, the categorization theory (Glucksberg, 2001, 2003, 2008; Torreano et al., 2005) was proposed to account for predicate metaphor processing. In the case of “*My job is a jail,”* the words “*job*” and “*jail”* are both exemplars of a superordinate category “*confining and unpleasant situation*.” Similarly, in the predicate metaphor “*The rumor flew through the office,”* the verb “*fly*” is a prototypical exemplar of the superordinate category “*fast travel*.” This process is a direct categorization (Glucksberg, 2001, 2003; Torreano et al., 2005) in which metaphorical meanings are achieved through direct abstraction from a superordinate category. However, Utsumi and Sakamoto (2011) doubted this explanation because the semantic structures of verbs are qualitatively different from those of nouns. Specifically, verbs involve events and action, and semantic relations entail rich elaborations and thematic roles, such as the hyponymy “To V1 is to V2 in some particular manner” (e.g., *to bend is to force a different shape or direction*). Nouns, however, refer to objects, and the semantic relations between nouns are mostly class inclusion, such as the superordination “A N1 is a N2” (e.g., *A canary is a bird*). Verbs are less likely to rely on hierarchical relations (Chen et al., 2008); therefore, the meanings of predicate metaphors may not be created via the superordinate categorization of the events that are evoked by verbs.

An intermediate entity is assumed to be activated by verbs before a figurative category of actions is finalized (Utsumi and Sakamoto, 2011). In the example *The rumor flew through the office*, the intermediate entity such as things that fly (e.g., *plane* or *bird*) or an imaginary scene of flying, may be evoked before an abstract meaning “*travel fast*” was created. In this case, understanding a predicate metaphor is a two-stage process with indirect categorization. An N400-LPC component observed in an ERP study (Obert et al., 2018) showed that semantic integration occurred during metaphor processing, and this result appeared consistent with the indirect view of categorization. However, these studies failed to specify the intermediate entity. Will the intermediate entity be the specific sensorimotor association from verbs or the prototypical agent of verb? When reading novel expressions such as *The taxes pinch the industry*, does one think of the act of pressing something between a thumb and a finger, a painful feeling, or a pair of shoes (a prototypical entity that pinches)? When reading familiar expressions such as *The media bent the truth*, does one directly retrieve the abstract meaning “*distort*” from long-term memory or rely on the concrete act of “*causing to curve*”? In this study, we assumed that if predicate metaphors are processed *via* intermediate entities, the sensorimotor aspects of verbs, then the pictures describing verb-related action can facilitate the understanding of metaphors.

It is well-known fact that understanding metaphors in a second or foreign language may impose great difficulty due to linguistic barrier. Thus, untangling how L2 learners process L2 metaphors could have important insights for those trying to teach a second language (Littlemore, 2009, 2019). There has been a great body of literature exploring relationships between metaphors processing and second language learning in terms of conceptual metaphor theory (Yasuda, 2010; Lu and Sun, 2017), metaphorical competence (Danesi, 1993; Littlemore and Low, 2006), metaphor awareness (Guo, 2007; Chen and Lai, 2012; Boers, 2013), imagery (Ifantidou and Hatzidaki, 2019) and individual differences (Johnson and Rosano, 1993; Wegner et al., 2020). Previous ERPs studies on bilingual metaphor processing found neural similarities and difference in metaphor processing between L1 and L2 (Dong, 2013; Park and Chung, 2013; Xue et al., 2014; Liu, 2016; Jankowiak et al., 2017, 2021; Wang, 2018). Although metaphoric meaning integration might be of similarity in L1 and L2, L2 learners require more intensive cognitive mechanism or continued effort in lexicon-semantic access and tended to adopt L1 neural pathway to process L2 metaphors. However, Citron et al. (2020) found L2 speakers were less affected by increasing metaphoricity than L1 speakers, suggesting metaphorical language is not necessarily engaging in L2 speakers. Yet this research did not take L2 proficiency into consideration since several neural studies found that L2 proficiency difference had an impact on neural activities in the brain areas responsible for novel metaphors (Dong, 2013; Liu, 2016).

Most literature of L2 metaphor comprehension focused on nominal metaphors and temporal-spatial metaphors, but lacked discussion upon predicate metaphors. Most ERPs studies described the processing mechanism at the neural level, but behavioral evidences that probe into the embodied features of L2 metaphor processing are insufficient. As predicate metaphors are cognitively challenging, how sensorimotor stimulation facilitates L2 predicate metaphor processing becomes an important concern. According to dual coding theory, verbal information (e.g., language) and non-verbal information (e.g., image) both contribute to cognition. From a bilingual point of view (Schnotz and Horz, 2010), lower-level L2 learners can perform better with two information sources (i.e., picture and text) while higher-level learners can perform well with only one source (i.e., text). In this sense, the embodiment effect of action-related pictures on predicate metaphor comprehension will be different among L2 learners of different levels.

In summary, it remains unknown whether and how predicate metaphor processing draws from the sensorimotor system, and the mechanism of intermediate entity during predicate metaphor processing has been unclear. The mixed findings in previous literature could be a result of difference in metaphor novelty, language proficiency and experimental paradigms. Given that English predicate metaphors are unfamiliar to Chinese L2 learners whose mother tongue has less of this construction, a second language perspective could help to reveal more about the embodied features of predicate metaphor processing. Taken together, we form the following questions: (1) Does the embodiment of verbs (i.e., the activation of sensorimotor aspects) influence L2 predicate metaphor processing? (2) How is the embodiment effect different between higher-level and lower-level Chinese L2 learners when they read English conventional predicate metaphors and novel metaphors? (3) Are L2 predicate metaphors comprehended via intermediate entity in an indirect process?

## Materials and Methods

### Participants

Participants were 100 paid native Chinese undergraduates who majored in English and have been learning English as second language since primary school. They were divided into two proficiency groups—higher-level group were 45 junior English majors (age range 21–23) who passed the *National Test for English Major, Band 8*), and lower-level group (age range 17–18) were 55 freshmen who scored above 120 on the *National College Matriculation English Test*. Informed consent was obtained from each participant prior to the experiment.

### Materials

The experiment contained three types of materials - target sentences, comprehension sentences and pictures. A total of 96 target sentences (see Table 1 and Supplementary file) were equally divided into four groups: conventional predicate metaphors (CMs), novel predicate metaphors (NMs) and their corresponding literal sentences (L-CMs, L-NMs). They were selected from previous studies (Cardillo et al., 2012; Desai et al., 2013) and two online dictionaries (https://www.thefreedictionary.com/ & https://dictionary.cambridge.org/).

**Table 1:** Examples of the four types of target sentences and comprehension sentences.

**Sentence type**	**Target sentences in English (L2)**	**Comprehension sentences in Chinese (L1)**
*CM* *L-CM* *NM* *L-NM*	*The newspaper bent the truth*. *The repairman bent the pipe*. *The tax pinched the industry*. *The man pinched my face*.	报纸歪曲了事实。 (bào zhǐ wāi q$\bar{\text{u}}$ le shì shí) 修理工掰弯管子。 (xi$\bar{\text{u}}$ lǐ g$\bar{\text{o}}$ng bāi wān guǎn zi) 税收损伤这个行业。 (shuì sh$\bar{\text{o}}$u sǔn shāng zhè gè háng yè) 男人捏了捏我的脸。 (nán rén niē le niē wǒ de liǎn)

*CM, conventional metaphor; NM, novel metaphor; L-CM & L-NM are the literal counterparts to conventional metaphors and novel metaphors, respectively. Underlined words indicate the English verbs*.

Metaphors were rated in terms of *novelty* ratings by another group of 42 undergraduate English majors using a 7-point scale (1 being very common, 7 being very novel). The novelty degree of novel metaphors was significantly higher than conventional ones [*t*_(1,46)_ = 15.991, *p* = 0.000]. All sentences were edited to fit experimental purposes - predicate metaphors use action verbs figuratively while literal sentences had the same verbs to describe an event. The subjects in metaphors and literal sentences were designed to induce either figurative or literal meanings of verbs. In the metaphor set, the subjects were the entities that make literal physical actions seemingly unlikely (e.g., *the newspaper* and *the tax*), which encourages participants to discover the non-literal aspects of metaphors and abstract figurative meanings. In contrast, the subjects in literal sentences are always persons or animate entities (e.g., *repairman* and *father*). Metaphorical and literal sentences were matched in terms of syntactic forms, subject animacy and abstraction levels. Three native English speakers proofread the sentences to guarantee naturalness.

Another 34 undergraduate English majors rated the linguistic proprieties of verbs and target sentences in Table 2 using a 7-point Likert scale. Critically, a significant difference emerged between conventional metaphors and novel metaphors in the *sentence familiarity* (*p* = 0.000) and *reading ease* (*p* = 0.005) since the novel metaphors were less familiar and more difficult to understand. There were no striking differences in the *frequency, familiarity, concreteness, imageability* and *relative embodiment* of verbs or in the number of words and letters in target sentences (see Table 2, all *p*s > 0.05). In addition, a separate 7-point rating on *verb familiarity* and *metaphor familiarity* was conducted by another 30 senior English majors and 30 freshmen English majors. There was no familiarity difference between higher-level learners and lower-level learners when they read the verbs from conventional and novel metaphors [verbs in *CMs*: M_Higher_ = 6.626, M_Lower_ = 6.564, *t*_(1,46)_ = 0.407, *p* > 0.05; verbs in *NMs*: M_Higher_ = 6.276, M_Lower_ = 6.178, *t*_(1,46)_ = 0.402, *p* > 0.05]. Both groups know the verbs well and the embodiment priming from action pictures would not be a result of differences in *verb familiarity*. No difference was found in the familiarity of conventional metaphors [*CM:* M_Higher_ = 5.034, M_Lower_ = 4.792, *t*_(1,46)_ = 1.200, *p* > 0.05]. Since familiarity is correlated with the frequency in the corpus and conventionality refers to the degree to which an expression is associated with figurative meaning (Al-Azary and Katz, 2021), the metaphor familiarity was adopted to represent conventionality. The ratings showed that CMs were largely considered conventional to both groups.

**Table 2:** Means of sentential and lexical properties of metaphors and literals.

**Properties**	***CMs***	***Literals***	***NMs***	***Literals***
	**M (SD)**	**M (SD)**	**M (SD)**	**M (SD)**
**Sentences**				
Novelty	2.229 (0.60)	1.178 (0.20)	4.915 (0.56)	1.349 (0.43)
Familiarity	5.482 (0.74)	5.724 (0.71)	4.187 (1.02)	5.701 (1.00)
Reading ease	6.221 (0.43)	6.344 (0.40)	5.777 (0.56)	6.091 (0.68)
Words	5.540 (0.66)	5.670 (0.82)	5.540 (0.66)	5.620 (0.82)
Letters	25.46 (2.02)	25.08 (3.12)	25.58 (3.40)	23.88 (3.84)
**Verbs**				
Letters	4.580 (0.97)		5.000 (0.93)	
Frequency	0.865 (0.80)		0.532 (0.62)	
Familiarity	6.607 (0.50)		6.227 (0.81)	
Concreteness	5.008 (0.95)		5.115 (0.52)	
Imageability	5.242 (0.67)		5.295 (0.49)	
Embodiment	6.085 (0.53)		5.798 (1.19)	

*CMs are conventional metaphors, and NMs are novel metaphors. Frequency values were taken from iWeb as average log per million frequency. The values for concreteness and imageability were converted from the MRC Psycholinguistic Database (Coltheart, 1981). The ratings for embodiment were based on relative embodiment (Sidhu et al., 2014)*.

Comprehension sentences were Chinese literal sentences that were either correct or wrong translation of English metaphors and literal counterparts. It was designed to encourage attentiveness and examine the comprehension accuracy. Participants should judge whether the Chinese translations were correct interpretations of English metaphors or literals. All Chinese sentences, translated and proofread by two proficient Chinese-English bilinguals, were matched in the *number of Chinese characters, interpretability* and *familiarity* (ratings assigned by 20 Chinese speakers, all *p*s > 0.05). The *yes/no* answers to comprehension tasks were equal in numbers and counterbalanced across metaphors, literals and fillers.

Pictures were the priming stimuli and were divided into action-related pictures and unrelated pictures. Related pictures carry action association and sensorimotor elements relevant to the verbs while unrelated pictures are irrelevant to action and depict static objects that have little motor association. These stimuli are based on a picture-naming study (Zhang and Yang, 2003) or other picture banks on the internet and then edited for experimental purposes. They are drawings in white lines with black ground and rated by 56 undergraduates in terms of *picture-concept agreement, visual complexity* and *familiarity*. There were no significant differences between action-related pictures and action-unrelated pictures (see Table 3, all *p*s > 0.05).

**Table 3:** Percentage of concept agreement, mean visual complexity and familiarity.

**Properties**	**Related pictures**		**Unrelated pictures**	
	***CMs***	***NMs***	***CMs***	***NMs***
	**M (SD)**	**M (SD)**	**M (SD)**	**M (SD)**
Concept agreement	0.909 (0.53)	0.910 (0.07)	0.870 (0.19)	0.859 (0.18)
Visual complexity	1.954 (0.28)	2.121 (0.32)	2.125 (0.72)	2.030 (0.66)
Familiarity	4.258 (0.26)	4.076 (0.38)	4.270 (0.63)	4.205 (0.58)

*CMs are conventional metaphors, and NMs are novel metaphors*.

### Procedure

**Figure 1:**
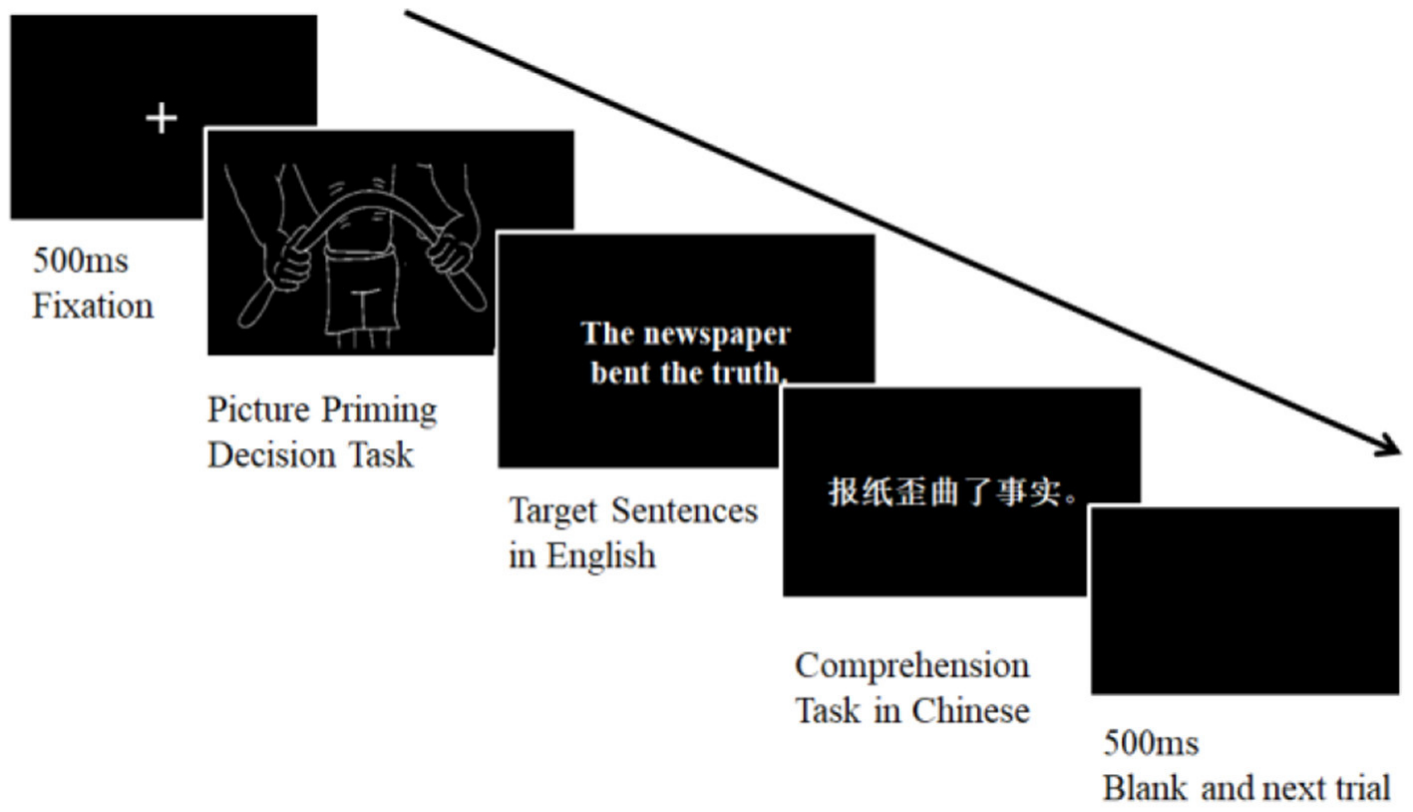
Procedure in the priming condition.

The sentences and pictures were presented visually on the screen (see Figure 1). After 500 ms fixation, a picture was presented for 2,000 ms when participants decided whether it depicted an action or static object as quickly as possible with a button press. Immediately, a target sentence followed, and participants were asked to press the space bar as soon as they understood it. Reaction times (RTs) were measured from the onset of target sentences until appropriate keys were pressed. Then, Chinese comprehension sentences appeared, and participants decided whether the translations were correct by pressing keys. Accuracy in comprehension tasks was recorded. Each participant read each target sentence, either primed with an action-related picture or unrelated picture, once. Metaphors, literals and priming conditions were counterbalanced. Eight practice trials were provided before the experiment.

## Results

After excluding participants with an error rate >15%, the behavioral data of 92 participants were valid. The average comprehension accuracy for higher-level group and lower-level group is 91 and 89%, respectively, indicating that the participants were engaged and attentive in the experiment. The analysis of reaction time was performed on the correct trials.

Table 4 shows the mean reaction time and mean accuracy. Concerning the data of metaphors, we found that conventional metaphors and novel metaphors were processed faster in the related priming condition than the unrelated priming condition (*CM:* M_Related_ = 3073.8 ms < M_Unrelated_ = 3289.3 ms, *p* = *0.000; NM:* M_Related_ = 3545.4 ms < M_Unrelated_ = 3955.4 ms, *p* = 0.000). Specifically, in the higher-level group, novel metaphors had a significant priming effect (M_Related_= 3328.9 ms < M_Unrelated_= 3661.1 ms, *p* = *0.002*) on reaction time while conventional metaphors and literals did not; in the lower-level group, the strongest priming effect was observed in the novel metaphors (M_Related_= 3712.0 ms < M_Unrelated_= 4181.9 ms, *p* = 0.000,), followed by the conventional metaphors (M_Related_= 3236.4 ms < M_Unrelated_= 3480.1 ms, *p* = 0.004). Therefore, the results of priming size seems to present a gradual facilitation on reaction time (*Diff*_*Lower*−*NM*_ = 469.9 ms, *Diff*_*Higher*−*NM*_ = 332.2 ms, *Diff*_*Lower*_*-*_*CM*_= 243.7 ms) across metaphor novelty, sentence type and proficiency groups.

**Table 4:** Mean (SD) reaction time (RTs) of target sentences and accuracy (ACC).

**Sentence type**	**Level**	**RTs (ms)**		***Diff*.**	**ACC (%)**		***Diff*.**
		**Related prime**	**Unrelated prime**		**Related prime**	**Unrelated prime**	
		**M (SD)**	**M (SD)**		**M (SD)**	**M (SD)**	
*CMs*	High	2862.5 (770.5)	3041.2 (918.1)	178.7	92.9 (7.7)	91.8 (7.3)	−1.0
	Low	3236.4 (862.4)	3480.1 (1135.8)	243.7	89.3(10.1)	88.8 (9.1)	−0.5
	Total	3073.8 (840.3)	3289.3 (1063.9)	205.5	90.8 (9.3)	90.1 (8.5)	−0.3
*NMs*	High	3328.9 (945.6)	3661.1 (1138.7)	332.2	91.3 (9.1)	89.4 (9.5)	−1.9
	Low	3712.0 (1158.1)	4181.9 (1334.9)	469.9	87.1 (9.9)	88.6 (9.5)	−1.5
	Total	3545.4 (1082.3)	3955.4 (1273.5)	410.0	88.9 (9.7)	88.9 (9.4)	0.0
*L-CMs*	High	2568.3 (797.3)	2673.6 (742.2)	105.3	93.6 (6.2)	90.3 (9.3)	−3.2
	Low	2913.7 (943.0)	2995.3 (934.6)	81.7	92.6 (7.6)	92.3 (7.9)	−0.3
	Total	2763.5 (894.7)	2855.4 (866.8)	91.9	93.0 (7.0)	91.5 (8.5)	−1.5
*L-NMs*	High	2607.3 (765.1)	2787.3 (777.0)	180.1	88.8 (9.9)	86.1 (9.4)	−2.7
	Low	2952.9 (818.1)	3102.8 (919.2)	149.9	86.4 (9.8)	86.4 (11.1)	0.0
	Total	2802.6 (809.7)	2965.6 (870.1)	163.0	87.5 (9.9)	86.3 (10.4)	−1.2

*CMs are conventional metaphor; NMs are novel metaphor; L-CMs & L-NMs are the literal counterparts to conventional metaphors and novel metaphors, respectively; High and Low refer to higher-level learners and lower-level learners, respectively. Diff. refers to the priming size between priming conditions*.

A four-way mixed ANOVA of *prime* (2) × *sentence type* (2) × *novelty* (2) × *proficiency* (2) is conducted on reaction times and accuracy by participants (F_1_) and items (F_2_). As shown in Table 5, significant effects on RTs were observed for all within-subjects variables: *prime* [F_1_ (1,90) = 46.789, *p* = 0.000, $\eta_{p}^{2}$ = 0.342; F_2_ (1,46) = 65.436, *p* = 0.000, $\eta_{p}^{2}$ = 0.587], *sentence type* [F_1_ (1,90) = 213.260, *p* = 0.000, $\eta_{p}^{2}$ = 0.703; F_2_ (1,46) = 66.387, *p* = 0.001, $\eta_{p}^{2}$ = 0.591], and *novelty* [F_1_ (1,90) = 126.12, *p* = 0.000, $\eta_{p}^{2}$ = 0.584; F_2_ (1,46) = 12.529, *p* = 0.001, $\eta_{p}^{2}$ = 0.214]. The between-subjects effect of *proficiency* was also significant [F_1_ (1,90) = 4.358, *p* = 0.040, $\eta_{p}^{2}$ = 0.046; F_2_ (1,46) = 5.676, *p* = 0.021, $\eta_{p}^{2}$ = 0.110]. Pairwise comparison showed that participants spent less time processing predicate metaphors in the action-related pictures condition than in the unrelated condition (M_Related_ = 3022.7 ms < M_Unrelated_ = 3240.4 ms, *p* = 0.000); literal sentences were processed much faster than metaphorical sentences (M_L_ = 2825.1 ms < M_M_ = 3438.0 ms, *p* = 0.000); all target sentences in the conventional conditions were processed faster than in the novel condition (M_CM_ = 2971.4 ms < M_NM_ = 3291.8 ms, *p* = 0.000). Higher-level group spent less time in reading target sentences than lower-level group did (M_High_ = 2941.3 ms < M_Low_ = 3321.9 ms, *p* = 0.040).

**Table 5:** Results of four-way ANOVA analysis on RTs.

**Effect**	***F* value**	***p***		$\eta_{p}^{2}$
Prime	46.79	0.000	^***^	0.342
Sentence type	213.3	0.000	^***^	0.703
Novelty	126.12	0.000	^***^	0.584
Proficiency	4.358	0.040	^*^	0.046
Prime x proficiency	0.342	0.560		0.004
Sentence type x proficiency	1.339	0.250		0.015
Novelty x proficiency	0.139	0.710		0.002
Prime x sentence type	6.032	0.016	^*^	0.063
Prime x novelty	5.208	0.025	^*^	0.055
Sentence type x novelty	56.73	0.000	^***^	0.387
Prime x novelty x proficiency	0.083	0.773		0.001
Prime x sentence type x novelty	1.089	0.299		0.012
Sentence type x novelty x proficiency	0.139	0.711		0.002
Prime x sentence type x novelty x proficiency	0.122	0.728		0.001

*** *p < 0.001 and*

* *p < 0.05*.

**Figure 2:**
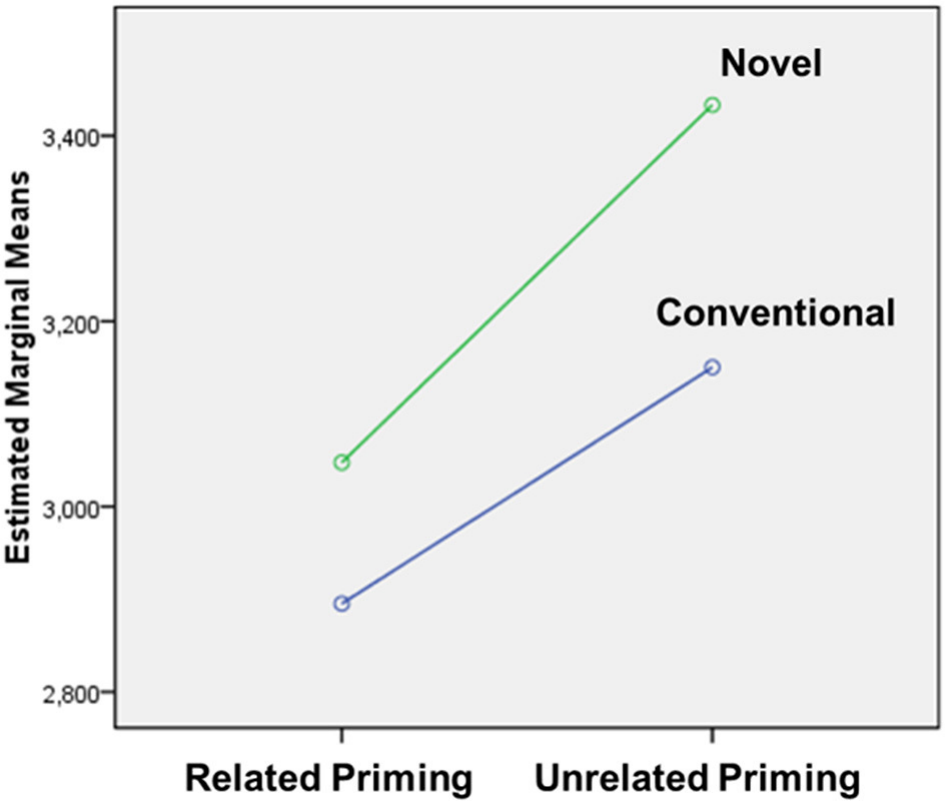
Interaction between prime and novelty.

The two-factor interactions were significant between *sentence type* and *novelty* (F_1_ = 56.732, *p* = 0.000, $\eta_{p}^{2}$ = 0.387; F_2_ = 17.843, *p* = 0.000, $\eta_{p}^{2}$ = 0.279), between *prime* and *sentence type* (F_1_ = 6.032, *p* = 0.016, $\eta_{p}^{2}$ = 0.063, F_2_ < 1), and between *prime* and *novelty* (F_1_ = 5.208, *p* = 0.025, $\eta_{p}^{2}$ = 0.055, F_2_ < 1). However, no interaction was observed with proficiency. Specifically, it took participants more time to read novel metaphors than conventional ones (M_CM_ = 3181.6 ms < M_NM_ = 3750.4 ms, *t* (1,183) = −13.719, *p* = 0.000), but no significant difference was found in the literal targets (M_L−CM_ = 2809.5 ms < M_L−NM_ = 2884.1 ms, *t* (1,183) = −1.796, *p* > 0.05). In the related priming conditions, literals were processed much faster than metaphor (M_M_ = 3309.6 ms < M_L_ = 2783.1 ms, *t* (1,183) = 10.496, *p* = 0.000) and in the unrelated conditions, the differences between sentences types were a bit greater (M_M_ = 3622.4 ms < M_L_ = 2910.5 ms, *t* (1,183) = 13.081, *p* = 0.000). Similarly, conventional targets were read faster than novel targets when they were primed with the pictures of actions (M_C_ = 2918.7 ms < M_N_ = 3072.4 ms, *t* (1,183) = −3.248, *p* = 0.001), but when the pictures were not action-related, the differences were slightly greater (M_C_ = 3174.0 ms < M_N_ = 3460.5 ms, t (1,183) = −6.983, *p* = 0.000). Figure 2 showed that the time gap between conventional targets and novel targets was smaller in the related-picture condition than in the unrelated conditions, suggesting the embodied priming effect was influenced by metaphor novelty.

Accuracy data presented a main effect for *prime* only with item analysis (F_2_ = 4.697, *p* = 0.035, $\eta_{p}^{2}$ = 0.093) but a stronger effect for *novelty* (F_1_ = 37.303, *p* = 0.000, $\eta_{p}^{2}$ = 0.239; F_2_ = 7.835, *p* = 0.007, $\eta_{p}^{2}$ = 0.146). *Proficiency* had little effect on accuracy (F_1_ = 3.032, *p* > 0.05; F_2_ < 1), suggesting that both groups of L2 learners did not differ greatly in the correct comprehension of predicate metaphors. No interaction effects were observed except one between *sentence type* and *novelty*, only with subject analysis (F_1_ = 9.283, *p* = 0.003, $\eta_{p}^{2}$ = 0.094; F_2_ = 1.272, *p* = 0.265, $\eta_{p}^{2}$ = 0.027).

We conducted two separate analyses to further explore embodiment effect on L2 predicate metaphor comprehension as two proficiency groups has demonstrated significant difference.

For higher-level learners, the three-way ANOVA of *prime* (2) × *sentence type* (2) × *novelty* (2) showed significant main effects of *prime* [F_1_ (1,39) = 17.356, p = 0.000, $\eta_{p}^{2}$ = 0.308; F_2_ (1,23) = 33.831, *p* = 0.000, $\eta_{p}^{2}$ = 0.595], *sentence type* [F_1_ (1,39) = 110.995, *p* = 0.000, $\eta_{p}^{2}$ = 0.740; F_2_ (1,23) = 35.055, *p* = 0.000, $\eta_{p}^{2}$ = 0.604] and *novelty* [F_1_ (1,39) = 46.184, *p* = 0.000, $\eta_{p}^{2}$ = 0.542; F_2_ (1,23) = 8.892, *p* = 0.007, $\eta_{p}^{2}$ = 0.279]. Yet different to the four-way ANOVA, only one interaction effect was found between *sentence type* and *novelty* [F_1_ (1,39) = 24.619, *p* = 0.000, $\eta_{p}^{2}$ = 0.387; F_2_ (1,23) = 11.921, *p* = 0.002, $\eta_{p}^{2}$ = 0.341]. The reaction time of conventional metaphors and novel metaphors differed greatly [M_CM_ = 2952 ms < M_NM_ = 3495 ms, *t* (1,79) = −8.409, *p* = 0.000], while that of L-CMs and L-NMs did not (M_L−CM_ = 2621 ms < M_L−NM_ = 2697 ms, *p* > 0.05).

Although a three-factor interaction was not observed, the reaction times of novel metaphors were greatly reduced when primed with related pictures, but those of conventional metaphors were not affected [see Table 4, *NM*: M_Related_ = 3328.9 ms < M_Unrelated_= 3661.1 ms, *t* (1,79) = −3.272, *p* = 0.002; *CM:* M_Related_= 3328.9 ms < M_Unrelated_ = 3661.1 ms, *t* (1,79) = −2.018, *p* > 0.05]. This result may be due to participants' higher L2 proficiency and familiarity with conventional metaphors. Higher-level L2 learners draw on the sensorimotor information of verbs when they comprehend unfamiliar predicate metaphors while the concrete aspects of verbs are not essential when they comprehend familiar metaphors. This seems to be consistent with the weaker version of embodied cognition and the graded feature of L2 embodiment. In addition, it suggests that novel predicate metaphors could be processed *via* the intermediate entity (i.e., concrete, sensorimotor aspects of verbs) in an indirect manner, whereas conventional predicate metaphors could be processed through direct abstraction (i.e. direct categorization), without sensorimotor entities emerging.

Accuracy data revealed a main effect of *prime* [F_1_ (1, 39) = 6.624, *p* = 0.014, $\eta_{p}^{2}$ = 0.145; F_2_ (1,39) = 2.483, *p* = 0.129], with more correct answers in the related priming condition. The effect of *novelty* was also present [F_1_ (1, 39) = 16.784, *p* = 0.000, $\eta_{p}^{2}$ = 0.301; F_2_ (1, 23) = 2.569, *p* = 0.123], but these effects were only found with subject analysis. There were more correct answers in the related priming condition (M_Related_= 91.6% < M_Unrelated_= 89.4%, *p* = 0.013), and less accuracy in the novel groups (M_CM_ = 92.1% < M_NM_ = 88.9 %, *p* = 0.000). Neither the main effect of *sentence type* nor any interaction effects were found in comprehension accuracy.

For lower-level group, the three-way ANOVA of reaction time revealed significant main effects of *prime* [F_1_ (1, 51) = 31.632, *p* = 0.000, $\eta_{p}^{2}$ = 0.383; F_2_ (1, 23) = 32.988, *p* = 0.000, $\eta_{p}^{2}$ = 0.589], *novelty* [F_1_ (1, 51) = 85.891, *p* = 0.000, $\eta_{p}^{2}$ = 0.627; F_2_ (1, 23) = 4.453, *p* = 0.046, $\eta_{p}^{2}$ = 0.162] and *sentence type* [F_1_ (1, 51) = 117.68, *p* = 0.000, $\eta_{p}^{2}$ = 0.698; F_2_ (1, 23) = 31.558, *p* = 0.000, $\eta_{p}^{2}$ = 0.578]. Although the results of main effects were similar between two groups, two significant interactions were found between *sentence type* and *novelty* [F_1_(1, 51) = 33.838, *p* = 0.000, $\eta_{p}^{2}$ = 0.399; F_2_ (1, 23) = 6.093, *p* = 0.021, $\eta_{p}^{2}$ = 0.209] and *prime* and *sentence type* [F_1_ (1, 51) = 6.060, *p* = 0.017, $\eta_{p}^{2}$ = 0.106; F_2_ (1, 23) = 1.195, *p* = 0.286]. Based on the data of metaphors, the interaction between *prime* and *novelty* was also significant [F_1_ (1, 51) =6.624, *p* = 0.013, $\eta_{p}^{2}$ = 0.115; F_2_ < 1]. The significant priming was found for both conventional and novel metaphors among lower-level learners (see Table 4, *CM:* M_Related_ = 3236.4 ms < M_Unrelated_ = 3480.1 ms, *p* = 0.004; *NM:* M_Related_ = 3712.0 ms < M_Unrelated_ = 4181.9 ms, *p* = 0.000), with greater facilitation for novel metaphors [*Diff*_CM_ =322.2 ms, *Diff*_NM_= 469.9 ms, *t* (1,51) = −2.574, *p* = 0.013]; whereas this effect was only significant in the novel metaphors among higher-level learners.

As for accuracy data, no main effect of *prime* was found (F_1_ < 1, F_2_ = 2.221, *p* > 0.05), which was different to the results of higher-level group. The main effect of *novelty* was great (F_1_ = 21.909, *p* = 0.000, $\eta_{p}^{2}$ = 0.300; F_2_ = 6.451, *p* = 0.018, $\eta_{p}^{2}$ = 0.219) as targets in the novel conditions are less familiar, but neither the effect of *sentence type* nor any interaction effects were observed.

## Discussion

Our findings reveal that the embodiment effect via picture priming was positive: L2 learners of both groups could comprehend predicate metaphors much faster when primed with action-related pictures. Metaphor novelty and L2 proficiency played significant roles during the process, with novel metaphors receiving more facilitation than conventional metaphors, and lower-level L2 learners gaining greater embodiment benefits than higher-level L2 leaners. In the following, we will further discuss how the embodiment of verbs influences L2 predicate metaphor processing when novelty and L2 proficiency are taken into consideration, and explore the possibility of intermediate entities as a process of indirect categorization.

### Graded Effect of Embodiment

The stronger view of embodied cognition holds that metaphors are strongly grounded in sensorimotor experience (Richardson et al., 2003; Gibbs, 2006b, 2013; Wilson and Gibbs, 2007; Bardolph and Coulson, 2014), but the weaker view argues that conventionalization or abstraction plays a crucial role–novel metaphors rely more on sensorimotor information about source concept, but their figurative meanings can become abstracted through repeated use and conventionalized. Sensorimotor information is gradually minimized or shed while core abstract meanings remain salient (Chen et al., 2008; Binder and Jeffrey, 2016; Zwaan and Rolf, 2016). According to a graded view of conceptual embodiment (Desai et al., 2011), conceptual representation consists of multiple levels of abstraction from sensorimotor inputs, but access to abstract meaning is subject to frequency or familiarity. For conventional metaphors, the representations existing in the brain are sufficient for adequate and fast processing. For novel metaphors, the stimulation of sensorimotor information can contribute to a better understanding of new metaphoric meaning.

We postulate that if the sensorimotor system does have an important role, the priming of action pictures should be outstanding on L2 predicate metaphor processing. This assumption was born out of embodied views, and the imagery account that the images play a role in prompting “emergent” properties, such as a feeling or experience, which are not directly linked with the literal meaning of the metaphor vehicle (Carston, 2010; Ifantidou and Hatzidaki, 2019). Our findings show a significant priming effect among higher-level L2 learners - novel metaphors benefited from embodiment effect of action pictures while conventional metaphors did not. However, lower-level L2 learners processed both types of metaphor much faster in the related-picture priming condition (see Table 4. *Diff*_*Lower*−*NM*_ = 469.9 ms, *Diff*_*Higher*−*NM*_ = 332.2 ms; *Diff*_*Lower*−*CM*_ = 243.7 ms), which suggests that lower level L2 brains may rely more on sensorimotor system and these concrete experiences may not be eliminated in the conventional metaphor processing. Such L2 evidence pertains to previous L1 studies (Desai et al., 2013; Jamrozik et al., 2016) and also echoes the graded view of conceptual embodiment.

From the bilingual perspective of dual coding theory (Soh, 2010; Paivio, 2014), verbal systems of bilinguals are separate, but their non-verbal systems are generally shared. It has been predicted that lower-level L2 learners will perform better with two information sources (e.g., pictures and text) while higher-level learners will perform well with only one source (e.g., text, Schnotz and Horz, 2010). This may explain why the priming benefits from pictures in this study were greatest when lower-level L2 learners read novel metaphors while little benefits were found for the higher-level group when they read conventional metaphors.

Studies have found an embodiment effect of pictures and gestures on word learning and memories (Mayer et al., 2015) or embodied representations in abstract words (Meteyard et al., 2012; Borghi and Zarcone, 2016; Repetto et al., 2017), yet for the first time we observe a graded priming effect of embodiment, subject to L2 proficiency levels and one type of sensorimotor input - pictures representing action. Since a simple opposition between the strong and weak view of embodiment does not capture the subtleties (Mahon and Hickok, 2016), a hybrid and graded view of the embodiment effect, regarding other factors, should be necessary to a sufficient theory.

### Metaphor Novelty and L2 Proficiency

Novel metaphors were found to be linked with creative thinking (Rataj et al., 2018; Wang et al., 2019) and require deeper processing. The career of metaphor theory (Bowdle and Gentner, 2005) holds that when first reading a novel metaphor, people prefer it to be expressed in simile form that involves an explicit comparison (e.g., A is like B), but it shifts into a metaphoric form (e.g., A is B) over the course of familiarization. Metaphoric abstraction derived from the comparison was stored in memory, and people categorize metaphors as members of an abstract category. Likewise, predicate metaphors follow such a natural history - evolving from novel to familiar metaphors until they lose most of their figurative meaning (Schmidt et al., 2010; Desai et al., 2011, 2013; Cardillo et al., 2012). In line with this, novel metaphors seem to be more susceptible to embodiment priming while conventional metaphors are not.

Regarding a second language, however, a different result is observed: higher-level L2 learners received a significant priming effect only in the novel metaphors whereas this effect was found in both conventional metaphors and novel metaphors among lower-level L2 learners. The results demonstrate a consistent effect that has been shown with native English speaker in a cross-modal lexical priming study (Al-Azary and Katz, 2021). They found low-familiar nominal metaphors (e.g., *Health is glass*) primed bodied-action associates (e.g., *break*) but not abstract associates (e.g., *fragile*), and low-familiar metaphors were hence processed via simulations, whereas high-familiar metaphors only primed abstraction associates. Given that metaphor novelty is relative to familiarity and language proficiency, lower-level L2 learners and novel predicate metaphors are more likely to receive greater priming effect by sensorimotor activation.

Graded salience hypothesis (Giora, 1997, 2003) proposed that metaphorical meanings that are highly salient can be accessed first. The novel metaphors in native languages and conventional metaphors in second languages were found to be activated in the same areas of right hemisphere, responsible for non-salient and novel meaning. In other words, metaphors that are conventional to native speakers could appear familiar to proficient L2 learners but unfamiliar to less proficient learners. This might be relevant to our findings as lower L2 proficiency may render less salience in conventional metaphors, embodied picture priming may pre-activate such salience and help less proficient L2 learners to understand both conventional and novel predicate metaphors.

Besides, a given concept is distributed over multiple modality-specific systems. For example, patients with motor system impairment shifted their reliance on motion-specific information to representations in the visual system (Barsalou, 2016). Then, in the case of lower-level L2 learners, they may benefit from seeing multiple sensorimotor information from visual pictures when they encounter difficulties in understanding predicate metaphors.

### Sensorimotor Elements as Intermediate Entity

There has long been debate as to whether metaphors are processed as abstractions or concrete representation. As an abstraction view, direct categorization (Torreano et al., 2005; Glucksberg, 2008) holds that categorization of predicate metaphors is a course of abstraction from verbs, directly attributing properties to topics. However, studies have shown that predicate metaphor processing may undergo indirect categorization via an intermediate entity (Utsumi and Sakamoto, 2007, 2011). In view of embodied cognition and simulation view, we postulate that this intermediate entity might be sensorimotor aspects of verbs, and concrete features of an action concept play a role in L2 predicate metaphor processing. The findings of the present study gave preliminary evidence to our assumption.

An intermediate entity could be the prototypical agent that performs an action literally referred to by a verb or an abstract action obtained by abstracting a verb, but previous studies failed to identify what was involved (Caillies and Declercq, 2011; Utsumi and Sakamoto, 2011; Obert et al., 2018). In the present study, the sensorimotor information has a priming effect on novel metaphors and lower-level L2 learners, but did not affect higher-level learners' comprehension on conventional metaphors. This suggests that lower-level learners process predicate metaphor *via* “sensorimotor” intermediate entity through an indirect way, whereas higher-lever leaners may go through direct categorization in which figurative meaning is understood via abstraction but concrete meanings of verbs are shed (Cardillo et al., 2012; Desai et al., 2013; Jamrozik et al., 2016). At this point, we proposed a hybrid model of categorization for L2 predicate metaphors, relative to language proficiency. This is also consistent to the simulation-abstraction hybrid view on the mechanism of nominal metaphors (Al-Azary and Katz, 2021).

Previous ERP studies that observed a two-stage time course can explain our results. Lai et al. (2019) found that metaphoric sense in predicate metaphor is based on concrete action semantics because the metaphoric-concrete effect was significant within an early time window (200–500 ms). Obert et al. (2018) observed not only an N400, an index of retrieval of an intermediate entity, but also a late positive effect - an integration process that distinguished metaphors from literal expressions. In the present study, if abstraction or direct categorization occurred first or the sensorimotor system played an ephemeral role, the embodiment priming effect of action pictures should have been little. In contrast, a significant effect was found. Despite being a behavioral experiment, the present study contributes to this line of literature by including L2 proficiency, metaphor novelty and a priming paradigm that can specify an intermediate entity. However, it remains to be seen what other types of intermediate entity (e.g., prototypical agents or auditory aspects of verbs) are involved and the findings of present study cannot rule out other processing model of predicate metaphors.

### L2 Predicate Metaphor Pedagogy

According to the revised hierarchical model (Kroll and Stewart, 1994), L1 has a stronger link and direct access to meaning, but L2 is more likely to require mediation *via* L1 translation until the bilingual acquired sufficient L2 proficiency. The meanings at conceptual levels are shared by L1 and L2, but the lexical levels are usually separated. For predicate metaphors, strengthening the link from an L2 verb to an L1 concept may help an L2 learner to retrieve abstract meanings faster. The dual coding theory of bilingual memory (Paivio and Csapo, 1969; Paivio, 2014) proposes that words are represented cognitively by verbal codes (e.g., text) and non-verbal codes (e.g., image), and semantic and sensorimotor information about concrete words is located in an imaginal system that is shared by L1 and L2. Therefore, action pictures that stimulate L2 mental images and deliver semantic-sensorimotor information of verbs are likely to mediate access to the L1 concept and thus facilitate L2 learners' comprehension of predicate metaphors.

In the classroom, L2 teachers can use concrete pictures describing the action of verbs or encourage a mental simulation of the imagery of the action. Since lower-level L2 learners will perform better with both verbal and non-verbal information (Schnotz and Horz, 2010), a special emphasis on multimodal instruction concerning verb embodiment or arousal of their sensor-motor-affective system becomes important for L2 learners' comprehension and memory. In addition, an explanation about intermediate entities, such as sensorimotor features and prototypical agents of verbs, could help them understand how concrete aspects of verbs are mapped to abstract meaning.

## Conclusion

Given the difficulties L2 learners experience with metaphoric language comprehension and the paucity of research on predicate metaphors, this study first explored how embodiment influences L2 learners' processing of predicate metaphors. The sensorimotor elements of action-related pictures provided a graded priming effect, which was modulated by L2 proficiency and metaphor novelty. The present findings not only have important theoretical implications for embodied cognition and categorization model for metaphor processing, but also offers practical suggestions for L2 acquisition and teaching.

However, there are limitations in the study. First, the familiarity rating for the verbs in the novel metaphors was relatively lower, although not at a significant level. Second, pictures may carry literal sense of the verb and the present study did not manage to exclude it from picture priming due to design difficulty. Third, the other types of intermediate entity were not addressed here. If the hypothesis about indirect categorization was correct, then it would be expected that the auditory aspects of verbs may serve as the intermediate entity to facilitate auditory predicate metaphor processing. Despite a body of fMRI studies on predicate metaphors, more ERP and eye-tracking studies are needed to investigate the time course, attention and other qualitative features. For example, participants tend to focus on subject-verb phrases and rate higher metaphoricity for subject-noun phrase sentences over object-noun phrase sets (Torreano et al., 2005). This indicated that more attention was given to the first half of the sentence. In summation, future studies should consider verb familiarity, conventionality, different types of intermediate entities and different experimental methods.

## Data Availability Statement

The original contributions presented in the study are included in the article/Supplementary Material, further inquiries can be directed to the corresponding author/s.

## Ethics Statement

Ethical review and approval was not required for the study on human participants in accordance with the local legislation and institutional requirements. The patients/participants provided their written informed consent to participate in this study.

## Author Contributions

YF contributes greatly to this study, responsible for conceptualization, methodology, software, experiment, formal analysis, and writing (original draft and editing). RZ was responsible for supervision, conceptualization, and writing (review and editing). Both authors contributed to the article and approved the submitted version.

## Funding

This study was supported by the Guangdong Provincial Philosophy and Social Science Foundation, China [Grant No. GD20CWY12].

## Conflict of Interest

The authors declare that the research was conducted in the absence of any commercial or financial relationships that could be construed as a potential conflict of interest.

## Publisher's Note

All claims expressed in this article are solely those of the authors and do not necessarily represent those of their affiliated organizations, or those of the publisher, the editors and the reviewers. Any product that may be evaluated in this article, or claim that may be made by its manufacturer, is not guaranteed or endorsed by the publisher.
